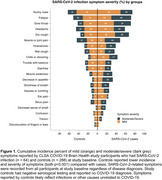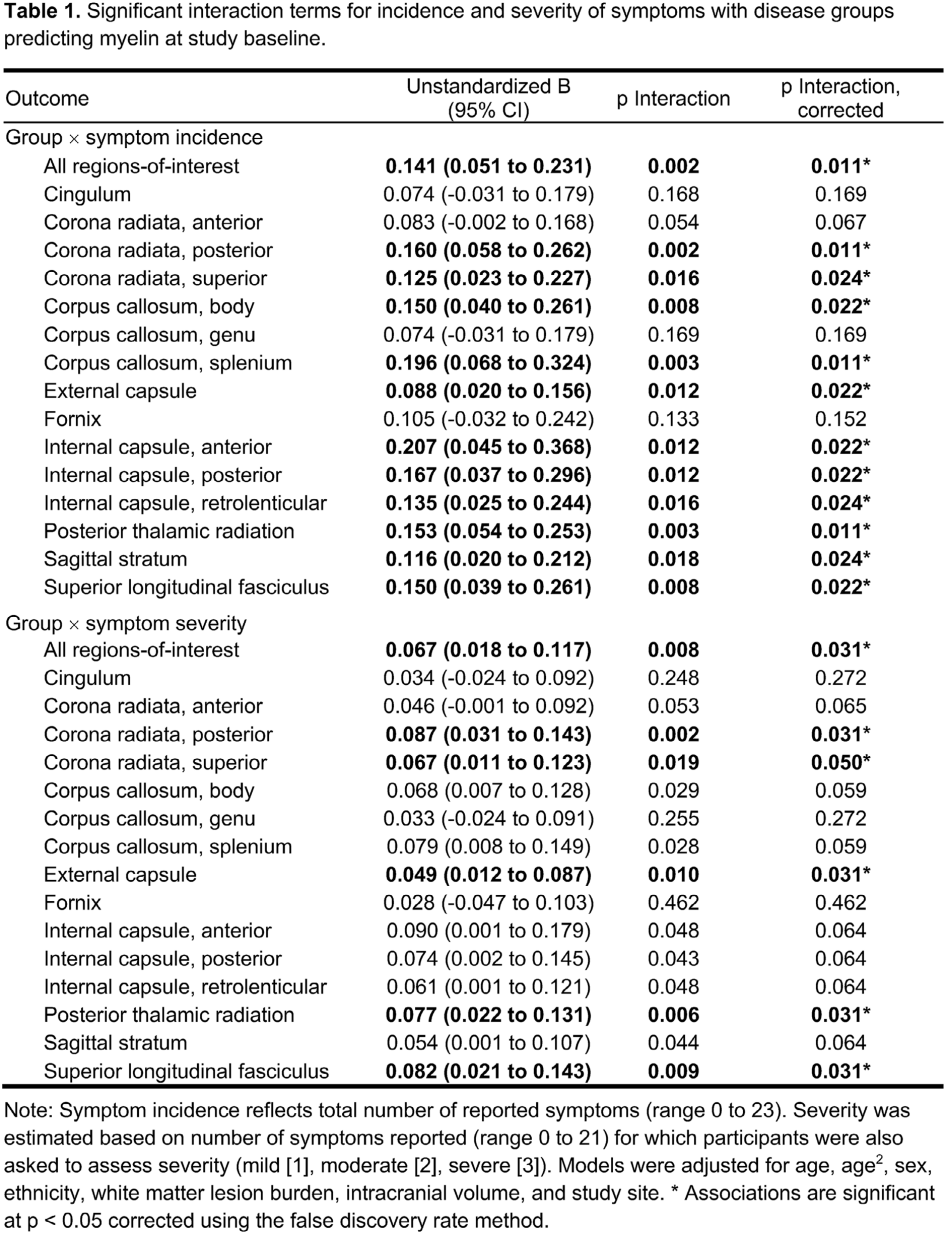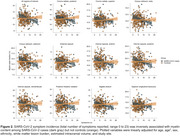# SARS‐CoV‐2 symptoms are negatively associated with myelin content in older individuals: Results from the Canadian Longitudinal Study on Aging COVID‐19 Brain Health Study

**DOI:** 10.1002/alz70861_108935

**Published:** 2025-12-23

**Authors:** Narlon C. Boa Sorte Silva, Ryan G Stein, Yi Gu, Chun Liang Hsu, Roger C. Tam, Marina Salluzzi, Cheryl R. McCreary, Walid A. Alkeridy, Kevin Lam, Alex L. MacKay, Shannon Kolind, Benoit Cossette, Lauren E. Griffith, David B. Hogan, Jacqueline M McMillan, Parminder Raina, Eric E. Smith, Teresa Liu‐Ambrose

**Affiliations:** ^1^ Concordia University, Montréal, QC Canada; ^2^ University of British Columbia, Vancouver, BC Canada; ^3^ The Hong Kong Polytechnic University, Hong Kong Hong Kong; ^4^ University of Calgary, Calgary, AB Canada; ^5^ King Saud University, Riaydh Saudi Arabia; ^6^ University of Sherbrooke, Sherbrooke, QC Canada; ^7^ McMaster University, Hamilton, ON Canada

## Abstract

**Background:**

Severe acute respiratory syndrome coronavirus‐2 (SARS‐CoV‐2) is the causative agent of COVID‐19 and has infected >700 million persons worldwide. Individuals infected with SARS‐CoV‐2 are at risk for cognitive decline and at higher risk of dementia compared with those diagnosed with other respiratory tract infections. Data from animal models suggest that SARS‐CoV‐2 infection triggers an overaggressive neuroinflammatory response resulting in myelin loss. Whether SARS‐CoV‐2 is associated with myelin loss in older individuals remains unknown.

**Methods:**

We investigated the impact of SARS‐CoV‐2 on myelin in older individuals from the Canadian Longitudinal Study on Aging COVID‐19 Brain Health Study who underwent brain MRIs. We included SARS‐CoV‐2 confirmed cases at baseline (2021‐2022) via positive serological testing or health care provider diagnosis. Non‐infected controls had negative serological testing and reported no COVID‐19 diagnosis. Myelin data were acquired via myelin water imaging using a 3D MRI gradient and spin echo sequence for T2 measurement. Myelin content was extracted from 16 regions‐of‐interest within the cerebral white matter. 3D T1‐weighted scans were acquired for registrations and to estimate intracranial volume. T2‐ and PD‐weighted scans were acquired for segmentation of white matter lesions. We performed cross‐sectional comparisons via analysis of covariance. Exploratory analyses were conducted to assess the association of SARS‐CoV‐2‐related symptom incidence and severity with myelin content by group. All models were adjusted for age, age^2^, sex, ethnicity, white matter lesion burden, intracranial volume, and study site.

**Results:**

We included 352 community‐dwelling individuals (SARS‐CoV‐cases, n= 64; controls, n=288). Their mean [SD] age was 65.26 (8.35) years, and 50.3% were female. There were no differences between SARS‐CoV‐2 cases and controls on myelin content across all regions‐of‐interest. Cases showed greater incidence (*p* <0.001) and severity (*p* <0.001) of symptoms compared with controls (Figure 1). Exploratory analysis revealed significant interactions between symptom incidence and severity with group after correcting for multiple comparisons (Table 1, p corrected < 0.05). *Post hoc* analysis showed that symptom incidence and severity were inversely associated with myelin in SARS‐CoV‐2 cases but not in controls across multiple regions‐of‐interest (Figure 2).

**Conclusions:**

Myelin loss may occur in older individuals who experienced greater incidence and severity of SARS‐CoV‐2 infection symptoms.